# Predictors of septic shock in obstructive acute pyelonephritis

**DOI:** 10.1007/s00345-013-1166-4

**Published:** 2013-09-15

**Authors:** Mitsuhiro Tambo, Takatsugu Okegawa, Toshihide Shishido, Eiji Higashihara, Kikuo Nutahara

**Affiliations:** Department of Urology, Kyorin University School of Medicine, 6-20-2, Shikawa, Mitaka, Tokyo 181-8611 Japan

**Keywords:** Acute pyelonephritis, Upper urinary tract calculi, Septic shock, Platelets, Albumin

## Abstract

**Purpose:**

Acute pyelonephritis (APN) with obstructive uropathy is not uncommon and often causes serious conditions including sepsis and septic shock. We assessed the risk factors for septic shock in patients with obstructive APN associated with upper urinary tract calculi.

**Methods:**

We retrospectively studied 69 patients with obstructive APN associated with upper urinary tract calculi who were admitted to our hospital. Emergency drainage for decompression of the renal collecting system was performed for empirical treatment in cases of failure of initial treatment and for severe cases. We assessed the risk factors for septic shock by multivariate logistic regression analysis.

**Results:**

Overall, 45 patients (65.2 %) underwent emergency drainage and 23 (33.3 %) patients showed septic shock. Poor performance status and the presence of diabetes mellitus (DM) in the septic shock group were more common than in the non-septic shock group (*p* = 0.012 and *p* = 0.011, respectively). The platelet count and serum albumin level in the septic shock group were significantly lower than in the non-septic shock group (*p* = 0.002 and *p* = 0.003, respectively). Positive rates of midstream urine culture and blood culture in the septic shock group were significantly higher than in the non-septic shock group (*p* = 0.022 and *p* = 0.001, respectively). Multivariate analysis showed that decreases in the platelet count (OR 5.43, *p* = 0.014) and serum albumin level (OR 5.88, *p* = 0.023) were independent risk factors for septic shock.

**Conclusion:**

Patients with obstructive APN associated with upper urinary tract calculi who have decreases in platelet count and serum albumin level should be treated with caution against the development of septic shock.

## Introduction

Complicated urinary tract infection (UTI) is an infection associated with organic or functional urinary tract abnormalities or an indwelling urinary catheter/device; it often fails to respond to conventional antimicrobial treatment. Complicated UTI with obstructive uropathy secondary to urinary calculi is not uncommon. Acute pyelonephritis (APN) with obstructive uropathy can progress to urosepsis and cause severe conditions such as septic shock and disseminated intravascular coagulopathy (DIC). It is accepted that the management of APN with obstructive uropathy is prompt decompression of the renal collecting system. The optional method of decompression is percutaneous nephrostomy or retrograde ureteral stenting. However, despite the emergent decompression for APN with obstructive uropathy, some cases can progress to septic shock and DIC. We assessed the risk factors for progression to septic shock in patients with obstructive APN associated with upper urinary calculi.

## Patients and methods

We conducted a retrospective medical chart review of all admissions to the Department of Urology, Kyorin University, Tokyo, Japan, that were due to APN with upper urinary tract calculi between January 1, 2006, and December 31, 2011. APN was defined as the presence of more than 5 white blood cells (WBCs)/high-power field (hpf) in a centrifuged urinary specimen, an isolated bacterial count of more than 10^4^ colony-forming units (CFU)/mL in the urine specimen, high-grade fever of more than 38 °C, and related characteristic symptoms. APN associated with surgical intervention was excluded in order to minimize potential bias for evaluating the risk factors in APN with upper urinary calculi. Antimicrobial treatment for initial empirical treatment was performed according to the antimicrobial treatment guidelines published by the Japanese Association for Infectious Disease and the Japanese Society of Chemotherapy that a cephalosporin, penicillin with a beta-lactamase inhibitor, an aminoglycoside, and a carbapenem, followed by oral antimicrobial treatment, are recommended [1]. The midstream and blood culture were performed in all patients, and antimicrobial susceptibility test also was examined. When susceptibility test of initial empirical treatment was resistant, antimicrobial treatment was exchanged to susceptible antibiotic. The duration of symptoms was defined as the time from the day of presentation to the day of intravenous antibiotic treatment initiation. Emergency drainage for the decompression of the renal collecting system was performed for empirical treatment in the case of initial failure and for severe cases (e.g., poor performance status, a markedly elevated leukocyte count, and C-reactive protein (CRP) level). We performed retrograde ureteral stenting for drainage as an initial trial and performed percutaneous nephrostomy in cases of initial failure or cases judged to present difficulty in inserting the ureteral stent (e.g., history of urinary tract abnormalities and severe hydronephrosis). The retrograde ureteral stenting was performed by fluoroscopic guidance using a rigid cystoscope with prior spinal anesthesia or transurethral administration of local anesthetic (if female). The 6Fr polyurethane JJ ureteral stents were used. The percutaneous nephrostomy was performed after sonographically guided renal puncture under local anesthesia. A guidewire insertion, fascial dilation, and 7Fr pigtail catheter placement were achieved by fluoroscopy.

Sepsis was defined as systemic inflammatory response syndrome (SIRS) (two or more of the following: body temperature >38.0 °C or <36.0 °C, heart rate >90 beats per minute, respiratory rate >20 breaths per minute or arterial CO_2_ tension <32 mmHg, white blood cell count >12,000/mm^3^ or <4,000/mm^3^ or immature neutrophils >10 %) and documented infection including culture or normally sterile body fluid positive for pathogenic microorganisms, or focus of infection identified by visual inspection. Severe sepsis was defined as sepsis and at least one sign of organ hypoperfusion or organ dysfunction, such as urinary output <0.5 mL/kg for at least 1 h, abrupt change in mental status, areas of mottled skin, platelet count <100,000 or DIC. Septic shock was defined as severe sepsis and one of the following: systemic mean blood pressure <60 mmHg (<80 mmHg if previous hypertension) after 40–60 mL/kg serum saline, or a need for dopamine >5 μg/kg per minute or norepinephrine (epinephrine) <0.25 μg/kg per minute to maintain mean blood pressure >60 mmHg (>80 mmHg if previous hypertension) [2]. Performance status was classified according to the World Health Organization performance status classification. The disease states including cardiovascular or neurologic diseases were described using the International Classification of Disease (ICD-10) definitions. Immunocompromised status included individuals who had a history of autoimmune diseases, liver cirrhosis or used immunocompromising drugs such as steroids. Either functional or structural abnormalities including hydronephrosis due to factors other than upper urinary tract calculi, urinary diversion such as ileal conduit, or the presence of an indwelling urinary catheter were categorized as urinary tract abnormalities. Estimated glomerular filtration rate (eGFR) (mL/min/1.73 m^2^) was calculated according to the following formula determined by the Japanese Society of Nephrology: 194 × serum creatinine (mg/dL)^−1.094^ × Age^−0.287^ × 0.739 (if female) [3]. Degree of hydronephrosis was graded based on the Society for Fatal Urology (grade 0: no calyx or pelvic dilation, grade 1: pelvic dilation only, grade 2: mild calyx dilation, grade 3: severe calyx dilation, and grade 4: calyx dilation accompanied by renal parenchymal atrophy) [4]. The low-grade hydronephrosis group included patients with grade 1 or 2 hydronephrosis, and the high-grade group included patients with grade 3 or 4 hydronephrosis.

### Statistical analysis

The variables in the different groups were compared using the Mann–Whitney *U* test. The independence of fitness of the categorical data was estimated by the chi-square test or Fisher’s exact test. Independent predictors of septic shock were determined using multivariate logistic regression analysis. Tests with a probability of less than 0.05 were considered as statistically significant. Statistical analyses were conducted using SPSS software (version 18.0).

## Results

Median age was 67 years. The male/female ratio was 0.53. In total, 23 patients (32.7 %) had poor performance status (2-4). Of the 69 patients, cases with underlying diseases including DM, cardiovascular or neurologic diseases, immunocompromised status, and urinary tract abnormalities numbered 17, 21, 8, and 4, respectively. Median serum creatinine and eGFR were 1.2 mg/dL and 40.9 mL/min/1.73 m^2^, respectively. Overall, 47 patients (68.1 %) showed a large shift of leukocyte count (<4,000 or >12,000/μL). Patients with decreased platelet count (<150,000/μL) and serum albumin level (<2.8 g/dL) numbered 17 and 15, respectively. In total, 51 patients (73.9 %) and 18 patients (26.1 %) were positive for midstream urine culture and blood culture, respectively. In addition, 50 patients (72.5 %) were positive for SIRS; however, none of the patients showed death related to the infection.

Most of the stones were presented in the ureter (79.7 %). The mean longest and shortest diameters were 11.1 and 7.2 mm, respectively. Overall, 33 patients (47.8 %) had high-grade hydronephrosis. Thirty-six patients (52.2 %) and 9 patients (13.0 %) underwent indwelling ureteral stenting (JJ stent) and nephrostomy, respectively, for decompression of the collecting system. The mean period from the initial treatment to decompression of the collecting system was 1.3 days. The male and history of urinary tract abnormalities were significantly more common in the nephrostomy group than in the ureteral stent group (*p* = 0.003 and *p* = 0.006, respectively). Serum creatinine, eGFR, and grade of hydronephrosis in the nephrostomy group are significantly higher than that in the ureteral stent group (*p* = 0.042, *p* = 0.025, and *p* = 0.005, respectively) (Table 1).

**Table 1:** Characteristics of patients who underwent ureteral stent and nephrostomy

Variables	Ureteral stent group		Nephrostomy group		*p* value
	*n*	%	*n*	%	
Patients	36		9		
Age (years)					
Median [range]	70 [33–98]		62 [49–81]		
Mean ± SD	66.6 ± 16.8		65.3 ± 12.0		0.831
Gender					
Male	8	22.2	7	77.8	
Female	28	77.8	2	22.2	0.003
Performance status					
0	19	52.8	6	66.7	
1	0	0.0	1	11.1	
2	7	19.4	0	0.0	
3	5	13.9	0	0.0	
4	5	13.9	2	22.2	0.109
Diabetes mellitus					
Negative	21	58.3	7	77.8	
Positive	15	41.7	2	22.2	0.249
Cardiovascular or neurologic disease					
Negative	23	63.9	7	77.8	
Positive	13	36.1	2	22.2	0.356
Immunocompromised status					
Negative	32	88.9	8	88.9	
Positive	4	11.1	1	11.1	0.691
History of urinary tract abnormalities					
Negative	36	100.0	6	66.7	
Positive	0	0.0	3	33.3	0.006
Serum creatinine (mg/dL)					
Median [range]	1.2 [0.4–7.8]		2.5 [1.1–7.0]		
Mean ± SD	1.6 ± 1.4		2.8 ± 1.7		0.042
eGFR (mL/min./1.73 m^2^)					
Median [range]	40.8 [4.4–112.3]		20.6 [7.1–42.3]		
Mean ± SD	44.1 ± 25.6		23.6 ± 11.3		0.025
Leukocyte counts (× 10^3^/μL)					
Median [range]	13.5 [1.8–47.0]		15.6 [9.1–27.9]		
Mean ± SD	15.9 ± 9.0		15.9 ± 6.4		0.999
C-reactive protein (mg/dL)					
Median [range]	12.9 [0.0–39.6]		18.5 [1.8–30.4]		
Mean ± SD	13.6 ± 9.3		16.0 ± 10.1		0.505
Platelet counts (× 10^4^/μL)					
Median [range]	15.3 [2.4–48.1]		15.6 [7.4–60.1]		
Mean ± SD	17.4 ± 9.8		20.2 ± 16.2		0.515
Serum albumin (g/dL)					
Median [range]	3.0 [1.9–4.5]		3.0 [1.8–4.3]		
Mean ± SD	3.0 ± 0.7		3.1 ± 0.9		0.821
Midstream urine culture					
Negative	7	19.4	2	22.2	
Positive	29	80.6	7	77.8	0.586
Blood culture					
Negative	25	69.4	6	66.7	
Positive	11	30.6	3	33.3	0.583
Hydronephrosis					
Low-grade	18	50.0	0	0.0	
High-grade	18	50.0	9	100.0	0.005
Laterality					
Right	19	52.8	3	33.3	
Left	17	47.2	6	66.7	0.252
Position					
Renal calyx or pelvis	1	2.8	0	0.0	
Pelvic ureteral junction	3	8.3	1	11.1	
Upper ureter	18	50.0	5	55.6	
Mid ureter	5	13.9	2	22.2	
Lower ureter	9	25.0	1	11.1	0.861
Size (mm) (mean ± SD)					
Longest diameter					
Median [range]	7.0 [4.0–35.0]		11.0 [8.0–19.0]		
Mean ± SD	8.7 ± 5.6		11.8 ± 4.0		0.131
Shortest diameter					
Median [range]	5.0 [2.0–15.0]		7.0 [5.0–12.0]		
Mean ± SD	5.7 ± 2.8		7.3 ± 2.0		0.106
Time to drainage (day)					
Median [range]	1.0 [1–3]		1.0 [1–4]		
Mean ± SD	1.2 ± 0.5		1.6 ± 1.0		0.155
Susceptibility test of initial antimicrobial treatment					
Resistant	5	13.9	3	33.3	
Susceptible	31	86.1	6	66.7	0.186
Time until susceptible antimicrobial treatment (day)					
Median [range]	1.0 [1–4]		1.0 [1–4]		
Mean ± SD	1.3 ± 0.8		1.8 ± 1.2		0.157
Duration of symptom (day)					
Median [range]	1.0 [1–9]		2.0 [1–6]		
Mean ± SD	1.9 ± 1.6		2.2 ± 1.6		0.618
Septic shock					
Negative	18	50.0	5	55.6	
Positive	18	50.0	4	44.4	0.530

*eGFR* estimated glomerular filtration rate

In total, 23 patients (33.3 %) showed septic shock. There was no significant difference in age between the septic shock and non-septic shock groups (*p* = 0.073). Performance status was poorer in the septic shock group than in the non-septic shock group (*p* = 0.012). DM was significantly more common in the septic shock group than in the non-septic shock group (*p* = 0.011). Cardiovascular or neurologic disease, immunocompromised status, and history of urinary tract abnormalities were not significantly different between the septic and non-septic shock groups (*p* = 0.107, *p* = 0.426, and *p* = 0.596, respectively). Of the 23 patients with septic shock, 22 (95.7 %) underwent drainage for decompression of the collecting system (ureteral stent or nephrostomy). There was no difference in the time from the initial treatment to decompression of the collecting system between the septic and non-septic shock groups (*p* = 0.263). With respect to laboratory data at the initiation of treatment, platelet count and serum albumin level in the septic shock group were significantly lower than in the non-septic shock group (*p* = 0.002 and *p* = 0.003, respectively); however, inflammatory reaction (leukocyte counts and CRP) and renal function (serum creatinine and eGFR) did not show significant difference between the septic and non-septic shock groups. The positive rates of midstream urine culture and blood culture in the septic shock group were significantly higher than in the non-septic shock group (*p* = 0.022 and *p* = 0.001, respectively); however, susceptibility test of initial antimicrobial treatment and time until susceptible antimicrobial treatment were not significantly different between the groups. There was no significant difference in stone demographics between the septic shock and non-septic shock groups (Table 2).

**Table 2:** Characteristics of patients with and without septic shock

Variables	Septic shock group		Non-septic shock group		*p* value
	*n*	%	*n*	%	
Patients	23		46		
Age (yr)					
Median [range]	73 [43–98]		65 [18–87]		
Mean ± SD	69.1 ± 13.8		62.2 ± 16.9		0.073
Gender					
Male	7	30.4	17	37.0	
Female	16	69.6	29	63.0	0.789
Performance status					
0	10	43.5	32	69.6	
1	1	4.4	3	6.5	
2	2	8.7	6	13.0	
3	3	13.0	4	8.7	
4	7	30.4	1	2.2	0.012
Diabetes mellitus					
Negative	11	47.8	37	80.4	
Positive	12	52.2	9	19.6	0.011
Cardiovascular or neurologic disease					
Negative	13	56.5	35	76.1	
Positive	10	43.5	11	23.9	0.107
Immunocompromised status					
Negative	19	82.6	42	91.3	
Positive	4	17.4	4	8.7	0.426
History of urinary tract abnormalities					
Negative	21	91.3	44	95.7	
Positive	2	8.7	2	4.3	0.596
Serum creatinine (mg/dL)					
Median [range]	1.8 [0.4–7.0]		1.1 [0.6–7.8]		
Mean ± SD	2.1 ± 1.5		1.4 ± 1.2		0.069
eGFR (mL/min./1.73 m^2^)					
Median [range]	26.5 [7.1–112.3]		44.1 [4.4–82.5]		
Mean ± SD	35.4 ± 27.6		45.5 ± 19.2		0.122
Leukocyte counts (× 10^3^/μL)					
Median [range]	15.7 [1.8–47.0]		13.7 [5.1–28.8]		
Mean ± SD	17.6 ± 10.3		14.8 ± 5.2		0.231
C-reactive protein (mg/dL)					
Median [range]	13.7 [0.2–39.6]		13.1 [0.0–41.7]		
Mean ± SD	15.0 ± 10.4		13.3 ± 9.4		0.506
Platelet counts (×10^4^/μL)					
Median [range]	11.6 [2.4–36.1]		21.1 [4.8–60.1]		
Mean ± SD	14.0 ± 9.2		22.3 ± 10.9		0.002
Serum albumin (g/dL)					
Median [range]	2.6 [1.8–4.3]		3.3 [2.1–4.5]		
Mean ± SD	2.8 ± 0.7		3.3 ± 0.6		0.003
Midstream urine culture					
Negative	2	8.7	16	37.2	
Positive	21	91.3	30	62.8	0.022
Blood culture					
Negative	11	47.8	40	87.0	
Positive	12	52.2	6	13.0	0.001
Hydronephrosis					
Low-grade	8	34.8	28	60.9	
High-grade	15	65.2	18	39.1	0.072
Laterality					
Right	9	39.1	24	52.2	
Left	14	60.9	22	47.8	0.444
Position					
Renal calyx or pelvis	1	4.4	5	10.9	
Pelvic ureteral junction	2	8.7	6	13.0	
Upper ureter	9	39.1	23	50.0	
Mid ureter	4	17.4	4	8.7	
Lower ureter	7	30.4	8	17.4	0.464
Size (mm) (mean ± SD)					
Longest diameter					
Median [range]	8.0 [4.0–35.0]		9.5 [2.0–80.0]		
Mean ± SD	9.8 ± 7.0		11.7 ± 12.3		0.415
Shortest diameter					
Median [range]	5.0 [2.0–15.0]		6.0 [2.0–39.0]		
Mean ± SD	6.0 ± 3.0		7.9 ± 7.6		0.140
Drainage					
None	1	4.4	23	50.0	
Ureteral stent	18	78.2	18	39.1	
Nephrostomy	4	17.4	5	10.9	0.001
Time to drainage (day)					
Median [range]	1.0 [1–3]		1.0 [1–4]		
Mean ± SD	1.2 ± 0.5		1.4 ± 0.7		0.263
Susceptibility test of initial antimicrobial treatment					
Resistant	6	26.1	6	13.0	
Susceptible	17	73.9	40	87.0	0.178
Time until susceptible antimicrobial treatment (day)					
Median [range]	1.0 [1–4]		1.0 [1–4]		
Mean ± SD	1.6 ± 1.0		1.3 ± 0.9		0.308
Duration of symptom (day)					
Median [range]	2.0 [1–9]		2.0 [1–4]		
Mean ± SD	2.3 ± 2.1		1.7 ± 0.8		0.245

*eGFR* estimated glomerular filtration rate

The continuous variables including platelet counts and serum albumin level were divided into two groups by receiver operating characteristic (ROC) curve. Multivariate logistic regression analysis showed that decreases in platelet count and serum albumin level were predictors of septic shock in patients with obstructive APN associated with upper urinary tract calculi (Table 3).

**Table 3:** Odds ratio for septic shock in relation to various factors by multivariate logistic regression analysis

Factor	OR	95 % CI	*p* value
Performance status			
0, 1	Ref		
2, 3, 4	1.52	(0.35–6.54)	0.574
Diabetes mellitus			
Negative	Ref		
Positive	3.34	(0.74–15.00)	0.116
Platelet counts			
15.0≦	Ref		
15.0>	5.43	(1.40–21.28)	0.014
Serum albumin			
2.8≦	Ref		
2.8>	5.88	(1.27–27.03)	0.023
Midstream urine culture			
Negative	Ref		
Positive	3.58	(0.38–33.33)	0.266
Blood culture			
Negative	Ref		
Positive	2.03	(0.42–9.80)	0.379

*OR* odds ratio, *CI* confidence interval

## Discussion

Urosepsis accounts for 20–30 % of all septic patients, and frequent causes of urosepsis are obstructive disease of the urinary tract, such as urinary stones, stenosis, and tumor [5]. In urosepsis, the severity depends mostly on the host response and local factors. Elderly patients and diabetic and/or immunosuppressed patients, such as patients with corticosteroids or immunosuppressant, transplant recipients and patients with AIDS, show reduced host defense. In addition, local factors, such as urinary tract calculi, obstructive uropathy, congenital uropathy, neurogenic bladder disorders, and endoscopic maneuvers, have an impact on the severity of urosepsis. Several studies confirmed that the presence of urinary tract obstruction in patients with bacteremic APN was associated with risk factors for the occurrence of septic shock [6, 7]. Obstructive uropathy of upper urinary tract complicated with infection should emerge to decompress the renal collecting system due to ureteral stent or percutaneous nephrostomy. Recently, a large population-level study showed that lack of decompression was associated with an increased odds ratio (OR 2.6, 95 % CI 1.9–3.7) of mortality in patients with sepsis and ureteral calculi [8]. In general, criteria for emergency drainage in patients with obstructive uropathy secondary to upper urinary tract calculi had been established in advance, based on the risk of sepsis, renal failure, and persistence of pain. Yoshimura et al. [9] reported that age, gender, and performance status were associated as risk factors for emergency drainage in patients with upper urinary tract calculi. In another study, age and a high CRP level were associated as useful parameters for deciding upon emergency drainage in patients with renal colic due to upper urinary tract calculi [10]. Regarding surgical decompression, Mokhmalji et al. [11] reported that percutaneous nephrostomy might be superior to ureteral stents for the diversion of hydronephrosis due to urinary calculi. A limitation of this study was that these procedures were performed under conscious sedation, and reasons for failure of the ureteral stenting were that it was intolerable due to the enlargement of the prostate and was performed on young men. In our institution, because the ureteral stenting was more convenient in daily life of patient than percutaneous nephrostomy, we performed as an initial trial for drainage of renal collecting system in patients with obstructive APN. The ureteral stenting in male patients was performed under spinal anesthesia not to be intolerable. However, 9 patients underwent percutaneous nephrostomy as an initial trail for drainage because of the history of urinary tract abnormalities and/or high-grade hydronephrosis. Difference of male/female ratio between ureteral stent and nephrostomy groups may be affected that history of urinary tract abnormalities showed significantly more common in male patients than in female patients (*p* = 0.032, data not shown).

In this study, we showed that decreases in platelet count and serum albumin level might be predictors of the development of septic shock in patients with obstructive APN. Severe sepsis often results in a decrease in platelets. It is thought that, although this is partly associated with the consumption of platelets by concurrent DIC, other factors also contribute. Platelets and neutrophils are actively involved in sepsis, and together, they cooperate to contribute to the inflammatory response. Neutrophil extracellular traps (NETs), an extracellular mechanism for microbial tapping and killing by neutrophils, are also emerging as an important part of the innate immune system in the defense against invading pathogens, and recently, it has been shown that platelets can mediate this mechanism. Both neutrophils and platelets have been shown to be recruited to the microvasculature of the liver and lung, leading to the production of NETs in models of sepsis [12]. Several studies demonstrated that many platelets localized in the liver and the lungs in septic model mice and septic humans [13, 14]. Consequently, accumulation of platelets in the liver and lung has a possible association with the decrease in platelets in sepsis.

Serum albumin is well known to decrease in response to inflammation. A decrease in serum albumin concentration can be a consequence of various factors, including increased protein catabolism and decreased hepatic synthesis [15], and lead to escape into the extravascular space because of increasing vascular permeability during the process of inflammation [16]. Bossink et al. [17] reported that, in febrile patients with a clinical infection, a low level of serum albumin was one of the predictive factors for shock development in univariate analysis.

Yamamoto et al. [18] reported that being elderly and the presence of paralysis might be risk factors for septic shock in patients receiving emergency drainage for APN with upper urinary calculi. The limitation of this study is the selection of subjects who had undergone emergency drainage for APN with upper urinary calculi. In our setting, one patient with septic shock could not undergo drainage because of a severe condition, and 24 patients did not undergo drainage because of improvement by initial empirical therapy. In a context similar to a daily clinical setting, we examined the risk factors for progression to septic shock in patients with obstructive APN associated with upper urinary tract calculi. However, our results are limited to a small number of enrolled patients and are from a retrospective study. Further studies are necessary using a larger prospective cohort.

## Conclusions

In patients with obstructive APN, decreases in platelet count and serum albumin level might be predictors of the development of septic shock. Patients with obstructive APN associated with upper urinary tract calculi who have these risk factors should be treated with caution.
